# Community-Dwelling Older Adults’ Intended Use of Different Types of Long-Term Care in China and Its Associated Factors Based on the Andersen Behavioral Model

**DOI:** 10.3390/ijerph191811626

**Published:** 2022-09-15

**Authors:** Run-Ping Che, Mei-Chun Cheung

**Affiliations:** Department of Social Work, The Chinese University of Hong Kong, Shatin, New Territories, Hong Kong SAR, China

**Keywords:** Andersen behavioral model, long-term care, community-dwelling older adults, associated factors, China

## Abstract

In light of the increased demand for long-term care services in China, there is an ongoing discussion on what factors contribute to older adults’ intended use of long-term care services. This study empirically recruited 239 community-dwelling older adults aged ≥60 years in China and explored factors influencing their intended use of four types of long-term care (basic life care, basic medical care, rehabilitation care, and psychological care) based on the Andersen behavioral model (i.e., predisposing characteristics, enabling resources, and need factors). The results showed that older adults were most likely to use psychological care. Age (as the predisposing characteristic) was the significant predictor of the intended use of four types of care. Regarding the intended use of basic life care, the enabling resources of marital status, household composition, income, as well as need factors of preference for the care setting, were influential. Moreover, income and need factors of self-rated physical health status were only two variables associated with the intended use of basic medical care. Concerning the intended use of rehabilitation care, household composition, income, self-rated physical health status, and preference for the care setting were significant predictors. The intended use of psychological care was influenced by enabling resources of marital status, household composition, and need factors of self-rated physical health status, preference for the care setting, and preference for the caregiver. These results can promote the sensitivity of policymakers and caregivers to the community-dwelling older adults’ intended use of long-term care and contribute to the delivery of appropriate care services by public policy.

## 1. Introduction

Since the beginning of the 21st century, the aging problem in China has become much more serious. By the end of 2018, the population of older adults aged ≥60 years in China was nearly 250 million, accounting for 17.9% of the total population. This will undoubtedly result in an upsurge in demand for long-term health care services, especially in some urban areas where the number of older adults increases faster. Additionally, the health status decline caused by aging renders the demands of older adults for long-term care services more urgent. By the end of 2018, the disability rate of older adults aged ≥60 years in China reached 11.8%. Moreover, among older adults who are disabled, 25% are entirely unable to take care of themselves [1]. To prepare the health care system for increased demand for long-term care services, it is necessary to identify older adults’ intention to use long-term care and provide them with proper care services.

Long-term care involves a broader range of services that can be summarized into four types, namely basic life care, basic medical care, rehabilitation care, and psychological care [2,3,4]. Basic life care refers to services that assist with daily activities, including feeding, bathing, dressing, toileting, medication, transportation, laundry, and other housekeeping tasks. Basic medical care includes nursing services that may require help from professional caregivers, such as wound care, intravenous therapy, medication administration, patient monitoring, pain control, and other nursing support. Rehabilitation care covers services that help older adults regain the abilities that they need for daily life. It may contain three aspects of support, namely physiotherapy, occupational therapy, and speech therapy. Psychological care aims to maintain the mental well-being of older adults. 

Currently, long-term care in China is provided for older adults with cognitive or physical impairments. Long-term care services mainly consist of two types: basic life care and basic medical care. By 2020, 426,000 older adults have been reported as long-term care service users [5]. Older adults can receive related services based on their preference in three kinds of settings: at home, in the community, and within nursing institutions [6]. Home care allows older adults to reside at home while being supported by in-home caregivers. Community care generally includes partial services that help older adults live independently in the community when in-home caregivers are inconvenienced and avoid social isolation, such as daytime care, or under arrangements made for respite care [7]. However, rehabilitation care and psychological care are neglected areas. This mismatch between the demand and supply of rehabilitation services has resulted in many people with disabilities not being able to access rehabilitation care [8]. According to an empirical study, 11.1% of Chinese older adults reported needing rehabilitation care, but less than 1% of them received related rehabilitation services in their community [9]. The same scenario occurs with psychological care. It has been reported that older adults in China suffer from a range of mental problems, with the prevalence of depressive symptoms being 23.6% in this population [10]. However, related services are not covered by the existing long-term care system. Additionally, the recognition rate of mental disorders in China is 21% (global average: 50%), making access to psychological care for older adults even more challenging [11]. Therefore, exploring older adults’ intention to use different types of long-term care can reflect which services are urgently needed. Related results can give insights to enhance current long-term care service design and delivery.

The use of long-term care for older adults is associated with multiple factors, which are normally integrated by the Andersen behavioral model. This model was initially proposed to describe and explain the intended or actual unitization of health services in the 1960s. According to the model, three domains of factors may influence health service use: predisposing characteristics, enabling resources, and need factors. Predisposing characteristics include people’s basic sociodemographic information, which suggests their biological needs for health services. Enabling resources is concerned with how someone can receive medical services and make use of them. Hence, this involves components such as income and marital status. For need factors, they highlight an individual’s perceived health status and other personal needs that could affect the use of health services [11]. Adopting the Andersen model to explore the intended use of long-term care can help us to understand the effects of different levels of factors. 

In previous studies, a range of factors that could explain the utilization of health services among older adults have been explored based on the Andersen model. The predisposing characteristics of age, gender, and education level were found to be significant predictors [12,13]. Moreover, enabling resources (such as marital status, living arrangements, and income) were reported to be associated with older adults’ needs [14,15]. In addition, physical health status, as the need factor, also strongly impacts older adults’ demand for care services [16,17]. In the Chinese context, studies on older adults’ long-term care service use and their influencing factors have not been researched in detail. Although limited studies have used the Andersen model to identify older adults’ preferences for the long-term care setting, the effects of three aspects of factors on different types of long-term care have not been mentioned [18,19]. Accordingly, this study incorporates older adults’ preferences for the care setting and the caregiver as independent variables into the need factors of the Andersen model to enrich the relevant theoretical results and expand the model. 

Based on the previous discussion, this study aims to answer the following questions: (1) What is the situation regarding the Chinese older adults’ intended use of different types of long-term care? (2) How do the factors of the Andersen model predict their intended use? The study examines the hypothesis that older adults’ intended use of different types of long-term care would be significantly influenced by the three domains of factors of the Andersen model. Regarding the two research questions, this study has two purposes. The first is to present and quantify the older adults’ intended use of long-term care. The second is to explore factors that influence the intended use of various types of long-term care services, trying to better understand the relationship between older adults’ intention to use long-term care and their characteristics. Based on these findings, more targeted care services can be provided for improving older adults’ well-being, and long-term care resources can be integrated more successfully.

## 2. Materials and Methods

### 2.1. Study Design and Participants

A multistage sampling method was adopted to select 252 participants in Changsha city in China. The first stage involved a random sample of 3 districts from a total of 7 districts in Changsha. In the second stage, 3 streets were randomly selected from each district. In the third stage, 2 communities were randomly selected from each street. In the final stage, 14 older adults will be randomly chosen as participants from each community. The inclusion criteria of participants were as follows: (1) Changsha residents with local households; (2) live in communities; (3) 60 years old or above. Older adults with visual, hearing, language, or cognitive impairments that affect their ability to fill in the questionnaire were excluded from the study.

### 2.2. Data Collection and Ethnic Issues

In 2019, a total of 252 questionnaire surveys (including 135 door-to-door interviews and 117 telephone surveys) were performed to collect data from eligible older adults. To help the participants feel at ease, they could freely choose either of the two interview methods and were allowed to be accompanied by their family members (or other people they trusted) when answering the questions. After filtering out invalid questionnaires with incomplete answers, 239 effective surveys were finally obtained. This study was approved by the Research Ethics Committee of Central South University, Changsha. All surveys were conducted with the permission of participants or their legal guardians, and the researcher explained the purpose of the study before moving on to the survey process. Informed consent was obtained from all participants. To ensure confidentiality, identifiers did not appear on any data.

### 2.3. Measurements

Based on the Andersen model, the independent variables of this study were summarized into three domains, as shown in Table 1. The predisposing characteristics included gender, age, and educational background. Enabling resources were expressed by marital status, household composition, and income per month. The need factors were identified as self-rated physical health status and participants’ preferences for the care setting and the caregiver.

As for the dependent variable, the scale to measure older adults’ intended use of long-term care was developed and validated by the researcher. Older adults were asked how likely they were to use each service. Their answers were rated on a 5-point Likert scale, ranging from very unlikely (1 point) to very likely (5 points), with the larger number representing a stronger intention. The scale contains 20 items that define services of basic life care, basic medical care, rehabilitation care, and psychological care. For these four types of care, which comprised 5, 7, 5, and 3 items, respectively, the sum of possible respective scores are 25, 35, 25, and 15.

The scale exhibited good validity and reliability. To ensure content validity, a panel of five experts with experience in nursing older adults was invited to score all items in terms of relevancy by using a 4-point ordinal scale (1 = not relevant, 2 = somewhat relevant, 3 = quite relevant, and 4 = very relevant). The content validity index (CVI) for each item was obtained by dividing the number of experts who judged the item as relevant (rating 3 or 4) by the total number of experts. The results indicated that the CVI for each item was >0.8, confirming that the scale had good content validity. Moreover, a principal components analysis was performed, and a stable four-factor solution with 20 existing items was revealed (factor loadings ≥ 0.50). By using Cronbach’s alpha, the internal consistency of the scale was 0.88. A pilot survey was conducted among 50 randomly selected eligible older adults in the Yuelu district of Changsha City, and a second survey was conducted on these older adults two weeks later. The results showed that the test–retest reliability for total scores was 0.89.

### 2.4. Data Analysis

The collected data were used to examine the hypothesis that older adults’ intended use of long-term care was significantly influenced by three domains of factors of the Andersen model. A descriptive statistical analysis was adopted to present the sociodemographic characteristics of the participants and the distribution of demand for each type of long-term care. Chi-square tests (χ^2^) were also performed to compare differences between the participants’ sociodemographic characteristics and their effects on need factors. Paired sample t-tests with composite scores were adopted to compare the use intention of older adults for different care types and services. The composite score of each care type was computed as the sum of corresponding question scores divided by the number of related questions. Hierarchical multiple regression analysis is an appropriate method for comparing the impact of different groups of variables. Hence, this was performed to predict how older adults’ intended use of four types of long-term care was influenced by the three domains of the factors proposed by the Andersen model. In this analysis, categorical independent variables were recoded and converted into dummy variables for regression analysis. The variables of predisposing characteristics were entered in the first block, while the variables of enabling resources and need factors were entered in the second and third blocks, respectively. To carry out the data analysis, SPSS version 25 was employed. In this study, the threshold for statistical significance was *p* < 0.01, with the consideration that the significance level was supposed to be set as a decreasing function of the sample size [20] and corrected for multiple comparisons.

## 3. Results

### 3.1. Sociodemographic Characteristics of the Samples

As shown in Table 1, over half of the samples were male (54.8%, *n* = 131). There was no significant difference in gender distribution among participants (*p* = 0.14). Furthermore, most of the participants were aged 70–79 years (41.8%, *n* = 100) and did not have a bachelor’s degree (87.4%, *n* =209). There were significant differences in the distribution of age (*p* < 0.01) and educational backgrounds (*p* < 0.001). Over 50% of the participants lived with others (such as their spouses and children) and were married. The differences in the distribution of marital status (*p* = 0.02) and household composition (*p* = 0.17) were not significant. The participants’ monthly income was concentrated around the levels of 2000–2999 RMB and 3000–3999 RMB. The difference in the distribution of income was significant. Nearly 40% of older adults thought they were in good or fair physical condition, while 67 (28.0%) claimed that they were in poor health. There was no significant difference in the distribution of their self-rated physical health status. Among the 239 participants, 40.6% (*n* = 97), 20.1% (*n* = 48), and 39.3% (*n* = 94) chose the home, the community, and the nursing home as their ideal settings to receive long-term care services, respectively. For caregiver preference, 44.8% (*n* = 107) believed that family members were ideal caregivers. Meanwhile, 20.7% (*n* = 49) and 34.5% (*n* = 83) of the participants preferred to be cared for by a home carer or a healthcare worker, respectively. The differences in the distribution of preference for the care setting and the caregiver were significant (*p* < 0.001).

### 3.2. The Effect of Predisposing Characteristics and Enabling Resources on Need Factors

According to the chi-square tests, there was a significant relationship between age and self-rated physical health status (*χ^2^*(4) = 66.14, *p* < 0.001). Specifically, the participants perceived themselves to be in poor physical health as they aged. It was also found that household composition was significantly associated with the preferences for the care setting (*χ^2^*(2) =27.67, *p* < 0.001) and the caregiver (*χ^2^*(2) =17.17, *p* < 0.001). Older adults who lived with others preferred to use care services at home and to be cared for by family members.

### 3.3. Intended Service Use of Long-Term Care

Table 2 presents the summarized descriptive analysis results of the 239 participants’ intended service use for different types of long-term care. The intended use of psychological care ranked highest among the needs of the four care types. The results of paired-sample t-tests of the four care types further indicated that the intended use of psychological care was significantly higher than the intended use of the other three types of care (*p* < 0.001). By using the same test to compare the intended use of services pertaining to psychological care, the results demonstrated that the intended use of group support was significantly higher than the other two services (*p* < 0.001).

### 3.4. Predictors of Intended Service Use of Long-Term Care

Table 3, Table 4, Table 5 and Table 6, respectively, present the hierarchical regression results of statistically significant predictors of the intended use of basic life care, basic medical care, rehabilitation care, and psychological care. Each table involves three models. The first model (Model 1) covered the independent variables of gender, age, and educational background. In the second model (Model 2), marital status, household composition, income, and ownership of medical insurance were added. The third model (Model 3) included newly added variables of self-rated health status and participants’ preferences for the care setting and the caregiver.

As shown in Model 1 of Table 3, the variables of predisposing characteristics significantly explained 18% of the variance in the intended use of basic life care (*F*(3, 235) = 16.62, *p* < 0.001). Age (*β* = 0.40, *t* = 6.75, *p* < 0.001) was a significant predictor of predisposing characteristics. Moreover, with increasing age, the intended use of basic life care also increased, which is consistent with reality. In Model 2, the addition of the variables of enabling resources resulted in significant changes in R^2^ of 20% (*F*(6, 232) = 23.53, *p* < 0.001). In addition, marital status (*β* = −0.19, *t* = −3.49, *p* < 0.001), household composition (*β* = 0.32, *t* = 5.75, *p* < 0.001), and income (*β* = 0.19, *t* = 3.57, *p* < 0.001) were all influential. As displayed in Model 3, preferences for care settings had an impact on older adults’ needs. Specifically, participants who preferred to be cared for in nursing homes (*β* = 0.36, *t* = 6.13, *p* < 0.001) required more services than those who preferred to be cared for at home. Overall, the final model explained 55% of the variance in the intended use of basic life care (*F*(12, 226) = 23.02, *p* < 0.001).

As shown in Model 1 of Table 4, the variables of predisposing characteristics significantly explained 41% of the variance in the intended use of basic medical care (*F*(3, 235) = 56.33, *p* < 0.001). Age (*β* = 0.63, *t* = 12.46, *p* < 0.001) was positively associated with the intended use of basic medical care. In Model 2, the variance in the intended use of basic medical care (explained by the addition of variables of enabling resources) was 4% (*F*(6, 232) = 28.80, *p* < 0.001). Income (*β* =.15, *t* = 2.80, *p* < 0.01) was the only variable of enabling resources that affected the intended use of basic medical care. Regarding Model 3, self-rated physical health status had a significant influence on the intended use of basic medical care. Older adults who described their health as poor (*β* = 0.22, *t* = 3.76, *p* < 0.001) or fair (*β* = 0.15, *t* = 2.65, *p* < 0.01) had a higher intention to use medical services than those who were in good health. Taken together, age, income, and self-rated physical health status explained 50% of the variance in the intended use of basic medical care (*F*(12, 226) = 19.19, *p* < 0.001).

From Model 1 of Table 5, it can be observed that the variance in the intended use of rehabilitation care explained by variables of predisposing characteristics was 33% (*F*(3, 235) = 39.87, *p* < 0.001). Age (*β* = 0.59, *t* = 10.42, *p* < 0.001) was a significant predictor. Regarding Model 2, the variables of enabling resources significantly additionally explained 12% of the variance in the intended use of rehabilitation care (*F*(6, 232) = 28.35, *p* < 0.001). Household composition (*β* = 0.28, *t* = 5.48, *p* < 0.001) and income (*β* = 0.14, *t* = 2.70, *p* < 0.01) were positively associated with the intended use of rehabilitation care. In Model 3, self-rated physical health status and preference for the care setting were influential. Older adults who described their health as poor (*β* = 0.22, *t* = 4.16, *p* < 0.001) or fair (*β* = 0.15, *t* = 3.60, *p* < 0.001) displayed a higher intention to use rehabilitation care. Compared with older adults who preferred to be cared for at home, those who preferred to be cared for in nursing homes (*β* = 0.24, *t* = 4.36, *p* < 0.001) required more rehabilitation care services. In summation, the final model revealed five variables that contributed significantly to the intended use of rehabilitation care, accounting for 58% of the variance in those needs (*F*(12, 226) = 26.04, *p* < 0.001).

As shown in Table 6, Model 1 yielded the result that the variables of predisposing characteristics explained 16% of the variance in the intended use of psychological care (*F*(3, 235) = 16.17, *p* < 0.001). Moreover, age (*β* = 0.38, *t* = 6.29, *p* < 0.001) had a positive association with the intended use of psychological care. From Model 2, it is evident that the variance in the intended use of psychological care (explained by the addition of the variables of enabling resources) was 12% (*F*(6, 232) = 13.94, *p* < 0.001). Marital status (*β* = 0.20, *t* = 3.51, *p* < 0.01) and household composition (*β* = 0.25, *t* = 4.29, *p* < 0.001) significantly predicted the intended use of psychological care. Concerning Model 3, the addition of variables of need factors resulted in an improvement in the model, with significant changes in ΔR^2^ of 18% (*F*(12, 226) = 16.49, *p* < 0.001). Self-rated physical health status, preference for the care setting, and preference for the caregiver were influential. Participants in poor health (*β* = 0.17, *t* = 2.77, *p* < 0.01) were more likely to need psychological services. Compared with older adults who wanted to be taken care of at home, those who preferred to be cared for in the community (*β* = −0.18, *t* = −3.42, *p* < 0.01) showed lower intention. Finally, older adults who hoped to be cared for by a health care worker (*β* = 0.23, *t* = 3.69, *p* < 0.001) displayed a higher intention to use psychological care than those who preferred to be cared for by family members. 

## 4. Discussion

By adopting the Andersen model, the intended use of long-term care and its associated factors were analyzed in Changsha city. The participants in this study were older adults, and more than 50% were male, which is consistent with the population demographics of Changsha city, where males account for 52.3% of the total population [21]. In terms of the age distribution of the elderly population, with the increase in life expectancy, the proportion of senior citizens (80 years old and above) in Changsha city rose from 7% in 1990 to 16.76% in 2019. Moreover, as suggested by this study, the education level of the elderly population in Changsha is generally not high, and more than 80% have not obtained a college degree or above [22]. In addition, most participants who lived with others chose their home as the ideal care setting and hoped to be cared for by family members, which is consistent with the findings of other studies conducted in the Chinese context [23,24]. Influenced by traditional Chinese family ethics, accepting health care from younger family members at home has been perceived as the mainstream of elderly care [25]. These close relationships between family members are an important guarantee of providing financial and physical support for older adults.

As demonstrated by the hierarchical linear regressions, older adults’ intended use of different types of long-term care was influenced by three domains of the Andersen model. Age was the only significant predictor of predisposing characteristics that had an impact on all types of care. Specifically, older adults’ intended use of long-term care services increased with age, which is consistent with the findings of previous studies [26,27]. Regarding the intended use of basic life care, the enabling resources of marital status, household composition, and income were significant determinants. Since some basic life care services are usually provided by spouses or family members, it is possible that unmarried older adults might not have a high intention to use these services compared to those who are currently married. Living alone increased older adults’ willingness to receive relevant services. One possibility for explaining this finding is that social isolation and a lack of contact with others will lead to a decline in health and an increase in the intended use of long-term care services [28]. In addition, higher incomes were positively associated with the intended use of care services. As mentioned in a previous study [29], older adults with higher income levels had stronger financial capacities to pay for long-term care. In addition, the association between preference for the care setting and the intended use of basic life care indicated that older adults who were inclined to be cared for in nursing homes had a higher intention than those who preferred to be cared for at home. This can be explained by the fact that older people may prefer to live in nursing facilities when their care needs are substantial and challenging to meet in home settings [30]. 

Concerning the intended use of medical care, income was the only influential factor in enabling resources. This finding provided further evidence that poor income can be a hindrance to health seeking and the utilization of care services [15,31]. However, the intended use of medical care was not influenced by other factors of the enabling resources. One possible explanation for this could be that the supply of medical care in China is relatively limited, and some parts of related services require additional fees. This makes it difficult for older adults to consider aspects other than economic status when expressing their intentions [6]. Among the need factors, the results revealed that poor physical status increased older adults’ intention to use medical care, which is supported by several studies [32,33]. 

Concerning the intended use of rehabilitation care, household composition and income of enabling resources were significant predictors. Moreover, older adults living alone intended more to use rehabilitation care services than those who lived with others, which was corroborated by a prior study indicating that living alone presented difficulties in creating a friendly environment for recovery [34]. Personal income was positively correlated with older adults’ needs, which is probably because some rehabilitation services are not covered by the resident health insurance scheme. Hence, older adults with higher incomes are more likely to access relevant services. Regarding the need factors, it is suggested that older people with poorer health have more demand for rehabilitation care compared to those with better health, which is in accordance with previous research [35,36]. Some chronic conditions that affect the health of older adults (such as dementia and cerebrovascular diseases) were not explored in this study. However, these have been proven to increase the probability of using rehabilitation care services [37]. The results also revealed that older adults who preferred to be cared for in nursing homes had a higher intention to use rehabilitation care than those who preferred to be cared for at home. This can be explained by the fact that some specialized rehabilitation services cannot be provided outside nursing homes.

For the intended use of psychological care, marital status and household composition of enabling resources were identified as significant predictors. Consistent with Pandey’s research [38], the results indicate that older adults who were not married required more psychological care services, which further illustrates that older adults with a spouse may have better mental health [39]. In line with previous studies [40,41], it was revealed that living alone was positively associated with the intended use of psychological care. This phenomenon can be explained by the fact that support from family members (or living partners) can enhance older adults’ psychological well-being [42,43]. All three need factors were reported as important determinants for predicting the intended use of psychological care. First, the results suggested that older adults with poor physical health status had a greater demand for psychological care, which was also confirmed by Bhandari and Paswan [44]. Furthermore, extensive evidence has indicated that age-related physical changes can cause problems for the psychological well-being of older adults, which can manifest as anxiety and loss of self-respect [26,45]. Moreover, it was revealed that older adults who preferred to be cared for in the community demanded less psychological care. This association could be interpreted by the fact that communities provide older adults with more opportunities for social interaction, which has been proven to play a role in relieving depression and anxiety [46,47]. The results also indicated that compared with older adults who preferred to be cared for by family members, those who hoped to be cared for by a health care worker were inclined to use psychological care. This is in accordance with the findings of previous research, which highlighted that family support can buffer the effects of stress and is regarded as the most helpful non-formal resource by older adults [48,49]. 

This study also provided new insights. For example, among the four types of long-term care, older adults exhibited the highest intention to use psychological care, while group support was the most needed service within psychological care. Before the 21st century, China’s central government prioritized restructuring the economy because people wanted their material needs to be met. It was not until the reform and opening up, when people’s living conditions improved, that older adults began to realize the importance of psychological well-being. In recent years, although China has gradually paid attention to improving the mental health of older adults, no relevant services have been provided in the long-term care system. In urban areas, psychological care services for older adults are mainly organized by non-governmental organizations (NGOs), such as the Red Cross, social enterprises, and school societies. In other words, the provision of these services is not universal. Some older adults may not have access to receive related services. With the release of the Mental Health Law and the National Mental Health Working Plan, more Chinese older adults may have perceived a need for psychological care. However, the problem of low awareness of mental health issues (and how they should be addressed) remains. According to the surveys conducted in Beijing and Shanghai, only 3.4% of respondents with psychiatric disorders asked for professional help in the previous year [50]. Similarly, in terms of an epidemiologic study conducted in Gansu, Qinghai, Shandong, and Zhejiang, only 8% of participants with mental disorders sought help from professionals [51]. Thus, continuing the promotion of psychological well-being in China is essential. 

Several strengths emerged in this study. First, numerous determinants were included and analyzed. Furthermore, this is one of the first studies to examine the predictors of the intended use of different types of long-term care in China. In addition, this study adds value to earlier research by introducing older adults’ preferences for the care setting and the caregiver into the need factor, which extends the current research model. However, there were some limitations to this study. First, the data were obtained from a questionnaire survey, and there may be a possibility of ambiguity about the direction of causal influence. Thus, further longitudinal studies should be conducted. Second, the samples for this study were only collected from one city in China, and the results may only reflect specific characteristics of that city. Third, other factors not included in this study (such as the number of children and mental health status) could also be significant predictors of long-term care utilization. Therefore, future studies should include these factors and examine whether they are influential. Furthermore, the relationship between intended use and actual use of long-term care services should receive attention. Exploring the differences between the intended and actual use of long-term care among older adults can provide a basis for service delivery and effective integration of care resources. Notably, geographic characteristics, such as the geographic distance between older adults’ homes and service providers and the distribution of long-term care agencies in communities, can also be considered when exploring the relationship between intended use and actual use of long-term care services.

## 5. Conclusions

In conclusion, all factors of the Andersen model are important for determining older adults’ intended use of different types of long-term care. Significant predictors that have already been mentioned in previous research (such as age, marital status, household composition, income, and self-rated physical health of need factors) were further confirmed in this study. Moreover, preferences for the care setting and caregiver were also proven to have an impact on the intended use of long-term care. The increasing intention of using psychological care highlights the existing public health problems. Therefore, mindful planning should be proposed to promote the mental health of older adults in China. 

Some practical implications for health policymakers can be drawn from the findings. First, it is necessary to assess the intended utilization of older adults for long-term care before providing services. In China, Activities of Daily Living (ADL) are currently used as the main basis for providing long-term care services. However, this standard clearly lacks a comprehensive measurement of older adults’ physical and mental status [52]. Therefore, composite assessment tools should be used to reflect the personal use intentions of older adults. In addition, the relevant assessment results can also serve as a reference for long-term service delivery and help avoid the waste of nursing resources.

Second, the power of community in caregiving should be valued. According to the results, older adults still rely heavily on family members for long-term care. However, with the successful implementation of the one-child policy and the rapid transformation and changes in society, family security functions are gradually weakening. The “421” (four grandparents, two parents, and one child) has become the typical family structure in China, which means that older adults have difficulties in relying on the support of family members alone to receive care services [53]. Therefore, in addition to family members, the community should also take advantage of its ability to integrate resources to become the organizer and provider of long-term care.

Finally, it is indispensable to strengthen mental health promotion for older adults. Although the participants in this study had a high intention to use psychological care, related services are not covered by the current long-term care system. Therefore, public health sectors should consider the implementation of policies and programs that facilitate the psychological care and protection of older adults. In addition, the results of some surveys conducted in China have indicated that older people have very limited experiences of dealing with their own mental problems. Therefore, knowledge of mental health issues should be popularized for older adults and their family members to avoid panic caused by emergencies. Moreover, as a social unit based on common geography, interests, and identity, the community plays a role in supporting older adults to maintain their psychological well-being. Community health care workers could help older adults promote social connectedness, and more community infrastructure should be developed to meet mental health care needs among older adults [54].

## Figures and Tables

**Table 1:** Sociodemographic characteristics of samples (*n* = 239).

	Grouping	Number	Frequency (%)
**Predisposing Characteristics**			
Gender			
	Male	131	54.8
	Female	108	45.2
Age (years)			
	60–69	86	36.0
	70–79	100	41.8
	80 or above	53	22.2
Educational Background			
	Below bachelor’s degree	209	87.4
	Bachelor’s degree or above	30	12.6
**Enabling Resources**			
Marital Status			
	Currently married	102	42.7
	Currently not married	137	57.3
Household Composition			
	Lives alone	109	45.6
	Lives with others	130	54.4
Income Per Month (RMB)			
	2000 or below	31	13.0
	2000–2999	67	28.0
	3000–3999	63	26.4
	4000–4999	37	15.5
	5000 or above	41	17.2
**Need Factors**			
Self-Rated Physical Health Status			
	Good	88	36.8
	Fair	84	35.2
	Poor	67	28.0
Preference for the Care Setting			
	Home	97	40.6
	Community	48	20.1
	Nursing home	94	39.3
Preference for the Caregiver			
	Family members	107	44.8
	Home carer	49	20.7
	Healthcare worker	83	34.5

RMB: Renminbi, the official currency of China.

**Table 2:** Participants’ Intended Use of Four Types of Long-Term Care Services (*n* = 239).

Intended Long-Term Care Service Use	$\;\bar{X\;}$	S.D.
**Basic life care**	**2.79**	**0.49**
Assistance in food preparation	2.63	1.03
Assistance in dressing	2.95	0.64
Assistance in toileting and excretion	2.85	0.58
Shopping	2.79	0.70
Transportation	2.75	0.58
**Basic medical care**	**2.85**	**0.59**
Instruction on taking medicines	2.80	0.61
Blood sugar testing	2.66	0.71
Blood pressure, breathing, and pulse measurement	2.86	0.67
Pain management	2.82	0.70
Medical injection	3.00	0.76
Oral hygiene measures	2.86	0.74
Wound care	2.92	0.87
**Rehabilitation care**	**2.88**	**0.62**
Post-hospital health care	2.80	0.69
Physical therapy	2.81	0.70
Assistance with equipment use	2.98	0.80
Create health files	2.89	0.77
Health education	2.93	0.86
**Psychological care**	**3.45**	**0.65**
Psychological consultation &counseling	3.38	0.72
Providing company	3.36	0.70
Group support	3.61	0.95

$\bar{X\;}$: Mean; S.D.: Standard Deviation.

**Table 3:** Hierarchical Regression Analysis of Factors Influencing the Intended Use of Basic Life Care.

	Model 1			Model 2			Model 3		
Variables	*β*	*t*	*p*	*β*	*t*	*p*	*β*	*t*	*p*
**Predisposing Characteristics**									
Gender									
Male	0.01	0.10	0.91	0.01	0.17	0.85	−0.03	−0.62	0.49
(ref: Female)									
Age	0.40	6.75	<0.001	0.30	5.51	<0.001	0.24	5.00	<0.001
Educational Background									
Below bachelor’s degree	−0.10	−1.71	0.09	−0.11	−2.01	0.05	−0.09	−1.90	0.04
(ref: Bachelor’s degree or above)									
**Enabling Resources**									
Marital Status									
Currently not married				−0.19	−3.49	<0.001	−0.13	−2.69	<0.01
(ref: Currently married)									
Household Composition									
Lives alone				0.32	5.75	<0.001	0.20	4.09	<0.001
(ref: Lives with others)									
Income Per Month				0.19	3.57	<0.001	0.19	3.83	<0.001
**Need Factors**									
Self-Rated Physical Health Status									
Poor							0.18	3.01	0.01
Fair							0.12	2.16	0.03
(ref: Good)									
Preference for the Care Setting									
Community							−0.05	−0.92	0.36
Nursing Home							0.36	6.13	<0.001
(ref: Home)									
Preference for the Caregiver									
Home Carer							−0.04	−0.09	0.82
Healthcare Worker							−0.05	0.42	0.40
(ref: Family members)									
Intercept	12.07 (*p* < 0.001)			10.96 (*p* < 0.001)			10.76 (*p* < 0.001)		
R^2^_adj_(ΔR^2^_adj_)	0.18			0.38 (0.20)			0.55 (0.17)		
F	16.62 (*p* < 0.001)			23.53 (*p* < 0.001)			23.02 (*p* < 0.001)		

*β*: Standardized Coefficient Beta; *t*: T statistic, R^2^_adj_: Adjusted R Square; F: F statistic.

**Table 4:** Hierarchical Regression Analysis of Factors Influencing the Intended Use of Basic Medical Care.

	Model 1			Model 2			Model 3		
Variables	*β*	*t*	*p*	*β*	*t*	*p*	*β*	*t*	*p*
**Predisposing Characteristics**									
Gender									
Male	0.08	1.64	0.10	0.08	1.76	0.08	0.07	1.57	0.12
(ref: Female)									
Age	0.63	12.46	<0.001	0.58	11.42	<0.001	0.52	10.45	<0.001
Educational Background									
Below bachelor’s degree	−0.10	−1.32	0.19	−0.04	−1.56	0.12	−0.07	−1.36	0.18
(ref: Bachelor’s degree or above)									
**Enabling Resources**									
Marital Status									
Currently not married				−0.07	−1.44	0.15	−0.05	−0.96	0.34
(ref: Currently married)									
Household Composition									
Lives alone				0.12	2.72	0.02	0.07	1.35	0.18
(ref: Lives with others)									
Income Per Month				0.15	2.80	<0.01	0.14	2.64	<0.01
**Need Factors**									
Self-Rated Physical Health Status									
Poor							0.22	3.76	<0.001
Fair							0.15	2.65	<0.01
(ref: Good)									
Preference for the care setting									
Community							−0.10	−1.87	0.06
Nursing Home							0.06	1.01	0.32
(ref: Home)									
Preference for the Caregiver									
Home Carer							0.05	1.05	0.29
Healthcare Worker							−0.05	−0.87	0.38
(ref: Family members)									
Intercept	13.48 (*p* < 0.001)			11.47 (*p* < 0.001)			11.78 (*p* < 0.001)		
R^2^_adj_(ΔR^2^_adj_)	0.41			0.45 (0.04)			0.50 (0.05)		
F	56.33 (*p* < 0.001)			28.80 (*p* < 0.001)			19.19 (*p* < 0.001)		

*β*: Standardized Coefficient Beta; *t*: T statistic, R^2^_adj_: Adjusted R Square; F: F statistic.

**Table 5:** Hierarchical Regression Analysis of Factors Influencing the Intended Use of Rehabilitation Care.

	Model 1			Model 2			Model 3		
Variables	*β*	*t*	*p*	*β*	*t*	*P*	*β*	*t*	*p*
**Predisposing Characteristics**									
Gender									
Male	0.10	1.86	0.06	0.10	1.97	0.05	0.06	1.33	0.18
(ref: Female)									
Age	0.59	10.42	<0.001	0.47	9.39	<0.001	0.41	8.98	<0.001
Educational Background									
Below bachelor’s degree	−0.02	−0.32	0.75	−0.02	−0.32	0.75	−0.01	−0.14	0.89
(ref: Bachelor’s degree or above)									
**Enabling Resources**									
Marital Status									
Currently not married				−0.11	−2.17	0.03	−0.06	−1.23	0.22
(ref: Currently married)									
Household Composition									
Lives alone				0.28	5.48	<0.001	0.18	3.79	<0.001
(ref: Lives with others)									
Income Per Month				0.14	2.70	<0.01	0.12	2.52	<0.01
**Need Factors**									
Self-Rated Physical Health Status									
Poor							0.23	4.16	<0.001
Fair							0.18	3.60	<0.001
(ref: Good)									
Preference for the Care Setting									
Community							−0.08	−1.69	0.09
Nursing Home							0.24	4.36	<0.001
(ref: Home)									
Preference for the Caregiver									
Home Carer							0.02	0.33	
Healthcare Worker							−0.01	−0.19	
(ref: Family members)									
Intercept	10.55 (*p* < 0.001)			9.32 (*p* < 0.001)			9.17 (*p* < 0.001)		
R^2^_adj_(ΔR^2^_adj_)	0.33			0.45 (0.12)			0.58 (0.13)		
F	39.87 (*p* < 0.001)			28.35 (*p* < 0.001)			26.04 (*p* < 0.001)		

*β*: Standardized Coefficient Beta; *t*: T statistic, R^2^_adj_: Adjusted R Square; F: F statistic.

**Table 6:** Hierarchical Regression Analysis of Factors Influencing the Intended Use of Psychological Care.

	Model 1			Model 2			Model 3		
Variables	*β*	*t*	*p*	*β*	*t*	*p*	*β*	*t*	*p*
**Predisposing Characteristics**									
Gender									
Male	0.09	1.55	0.12	0.06	1.19	0.24	−0.03	−0.06	0.95
(ref: Female)									
Age	0.38	6.29	<0.001	0.26	4.99	<0.001	0.23	4.50	<0.001
Educational Background									
Below bachelor’s degree	−0.10	−1.62	0.11	−0.11	−1.91	0.06	−0.01	−2.07	0.04
(ref: Bachelor’s degree or above)									
**Enabling Resources**									
Marital Status									
Currently not married				0.20	3.51	<0.01	0.27	5.26	<0.001
(ref: Currently married)									
Household Composition									
Lives alone				0.25	4.29	<0.001	0.13	2.46	<0.01
(ref: Lives with others)									
Income Per Month				0.19	3.30	0.10	0.12	2.29	0.02
**Need Factors**									
Self-Rated Physical Health Status									
Poor							0.17	2.77	<0.01
Fair							0.07	1.28	0.20
(ref: Good)									
Preference for the Care Setting									
Community							−0.18	−3.42	<0.01
Nursing Home							0.16	2.52	0.01
(ref: Home)									
Preference for the Caregiver									
Home Carer							0.11	1.99	0.04
Healthcare Worker							0.23	3.69	<0.001
(ref: Family members)									
Intercept	8.74 (*p* < 0.001)			7.41 (*p* < 0.001)			7.68 (*p* < 0.001)		
R^2^_adj_(ΔR^2^_adj_)	0.16			0.28 (0.12)			0.46 (0.18)		
F	16.17 (*p* < 0.001)			13.94 (*p* < 0.001)			16.49 (*p* < 0.001)		

*β*: Standardized Coefficient Beta; *t*: T statistic, R^2^_adj_: Adjusted R Square; F: F statistic.

## Data Availability

The data presented in this study are available on request from the corresponding author. The data are not publicly available due to privacy/ethical restrictions. There is confidential information that could compromise the anonymity of research participants as potential indirect identifiers, e.g., the community of residence of respondents.
